# Features spaces and a learning system for structural-temporal data, and their application on a use case of real-time communication network validation data

**DOI:** 10.1371/journal.pone.0228434

**Published:** 2020-02-06

**Authors:** Guido Schwenk, Ben Jochinke, Klaus-Robert Müller

**Affiliations:** 1 Machine Learning Group, Technische Universität Berlin, Berlin, Germany; 2 P3 group, Am Kraftversorgungsturm, Aachen, Germany; 3 Department of Brain and Cognitive Engineering, Korea University, Anam-dong, Seongbuk-gu, Seoul, Korea; 4 Max Planck Institute for Informatics, Stuhlsatzenhausweg, Saarbrücken, Germany; Universitat de Valencia, SPAIN

## Abstract

The service quality and system dependability of real-time communication networks strongly depends on the analysis of monitored data, to identify concrete problems and their causes. Many of these can be described by either their structural or temporal properties, or a combination of both. As current research is short of approaches sufficiently addressing both properties simultaneously, we propose a new feature space specifically suited for this task, which we analyze for its theoretical properties and its practical relevance. We evaluate its classification performance when used on real-world data sets of structural-temporal mobile communication data, and compare it to the performance achieved of feature representations used in related work. For this purpose we propose a system which allows the automatic detection and prediction of classes of pre-defined sequence behavior, greatly reducing costs caused by the otherwise required manual analysis. With our proposed feature spaces this system achieves a precision of more than 93% at recall values of 100%, with an up to 6.7% higher effective recall than otherwise similarly performing alternatives, notably outperforming alternative deep learning, kernel learning and ensemble learning approaches of related work. Furthermore the supported system calibration allows separating reliable from unreliable predictions more effectively, which is highly relevant for any practical application.

## Introduction

Sequences of structural and temporal data combine properties of complex symbolic sequences and multi-variate time series, in that a single sequence *s* of length *r* has the format *s* = [(*t*(*e*_1_), *e*_1_), …, (*t*(*e*_*r*_), *e*_*r*_)], i.e. each event *e*_*i*_ occurs at timestamp *t*(*e*_*i*_) within *s*, s.t. *t*(*e*_*i*_) = *t*(*s*)_*i*_. Additionally each sequence *s* can have a label *y*(*s*). When trying to process such data with temporal properties (i.e. semantically relevant, quantitative time intervals of varying length between individual events) and structural properties (i.e. a semantically relevant order and context of events and their represented behavior), research is often faced with different problems, like varying sequence lengths and the lack of feature spaces that allow representing both temporal and structural properties sufficiently well. With suitable feature spaces, processes that rely on such data can be better analyzed and represented, consequently allowing the application of a wide range of learning methods on those features.

This is true for all processes that create time-dependent structured data, like multi-layer protocol-based network communication, whose data can be recorded in real-time by logging systems. This allows the analysis of its structural-temporal data, utilizing its structural properties (e.g. protocol state-machine behavior) and its temporal properties (e.g. response timings) to continuously improve the quality of the respective communication service. The problem becomes even more complicated when analyzing the data of multi-directional real-time communication setups, as in video conferencing systems, in cloud infrastructures [1] or even in industrial infrastructure [2] networks. In those cases the temporal and structural properties are contained in multiple interacting event sequences, and the temporal properties are of vital relevance for the service quality of the system.

To be able to apply machine learning methods to solve the different types of problems occurring on such data (e.g. classification or prediction), specific feature spaces are required that properly represent those structural and temporal data properties and allow projecting sequences of arbitrary length onto feature vectors of homogenous length. To achieve this we analyze the structural and temporal properties of such highly time-dependent multi-client sequence data, and propose a new combined feature space which integrates structural and temporal properties in a novel way and allows for an equivalent length of the projected sequences, simplifying the subsequent application of various learning methods. We also show with an extensive competitive evaluation and statistical analysis how this feature space succeeds in this task.

As practical use case for structural temporal data we focus on bi-directional multi-client real-time mobile communication data, used to solve the specific problem of detecting and predicting known sequence classes on data of both known and unknown sequence classes. On this data all of the previously mentioned properties are relevant, i.e. the order and context of the events are class specific and relevant, as are both the individual and the interacting sequences of both clients, as well as the temporal properties represented in the contained timestamps and their sub-sequences. For this use case we introduce and evaluate a system for the automatic classification of failures of communication sequences between mobile clients. This system allows supporting or replacing the expensive manual classification usually handled by domain experts. We conduct a detailed analysis of the practical implications and requirements, especially on how to calibrate the system for a high precision while achieving a reasonable effective recall. This property is one of the main motivations of this manuscript and highly relevant in practice, as only highly reliable predictions allow rigorous consecutive decisions. On this system we comparatively evaluate the classification performances when using the proposed feature spaces with baseline, as well as competitive deep learning, kernel learning and ensemble methods from the fields of process mining and sequence classification, allowing to draw implications on their general suitability and their individual advantages and disadvantages.

While we are focusing our analyses on the specific area of mobile communication, the proposed system for the detection and prediction of pre-defined classes of sequential data, as well as the proposed feature spaces can be applied in all areas working with structural temporal data, specifically for problems that require the incorporation of real-time or multi-client system properties. This enables more precise classifiers and reduces the amount of required manual analysis, whose expensive cost would otherwise prevent the scaling of such a classification system to large scale data sets.

Summarizing the primary objectives, we aim to go beyond existing approaches of process mining and sequence learning by proposing a combination of structural and temporal features and their integration into a system for multi-class detection and prediction. As such the contributions of this paper are the following:

1. We propose a feature space for structural sequential data, which combines the use of both structural and temporal properties and integrates contextual positional variance in a novel way
2. We show the advantageous classification performance of the proposed feature space against feature spaces and learning methods commonly used in comparable process mining and sequence classification publications, including a baseline combined feature space
3. We further strengthen these analysis results with a significance analysis of the classification performances
4. We propose a combined detection and prediction system for multi-class failure classification of structural temporal data of mobile communication, with an additional focus on the calibration of the results’ reliability, which is highly relevant in practical applications

This paper is structured as follows: This Introduction is followed by a discussion of the Related Work. Then the use case and the utilized datasets are discussed in the Section Use Case Description, followed by the introduction of the relevant Structural and Temporal Feature Spaces. Afterwards the System Layout of the proposed sequence class detection and prediction are described, including the utilized learning methods. This is followed by the Evaluation and a statistical analysis of the classification system and the introduced feature spaces, before finally reaching the Conclusion.

## Related work

We are interested in analyzing and evaluating features spaces representing the unique properties of sequences of structural, temporal data, whose inter-event *structural properties* have a semantically relevant relation to each other. We do this with a focus on mobile communication data, as an example of real-time protocol-based *network communication* data, where both the raw data logs and logs of additional dynamic analysis allow representing the contained structural dependencies. Further details on mobile communication protocol behavior can be found in [3]. While similar analyses have been done for network communication, as e.g. in [4–11], most research focusses on the structural data properties, and less on the temporal properties relevant for the real-time execution of such processes.

We are using different types of features to represent the structural as well as the temporal data properties within the feature spaces. Specifically we are relying on token *n*-grams, i.e. sequences of *n* arbitrary tokens. This feature representation is similar to spectrum kernels [12] and originates in the field of natural language processing [13–16], but has also been extended to network communication [17–21]. However, we are extending this structural feature type by including additional temporal information, and by also integrating a wider context for each token *n*-gram, an idea similar to the integration of additional context information as introduced in [22] for the CBOW (continuous bag of words) and Skip-gram models.

This makes our research unique but also highly relevant for commercial processes, where such approaches are still highly sought after, e.g. in the form of *process mining* [23]. In this field different objectives are solved on business analytics data, which often comes in a format similar to our, i.e. consisting of events and timestamps. However, research in this field did so far not address multi-class classification problems, but focuses primarily on very narrow objectives, which do not require a combination of those different properties that we are interested in. As such [24] are using Markov Classifiers to predict the time remaining to completion of a business case. This objective is also addressed by the system proposed in [25], which utilizes Naive Bayes Classification and Support Vector Regression approaches, and in [26] which uses Long Short Term Memory neural networks (LSTM). The verification of linear temporal logic (LTL) compliance is approached in [27] by using Decision Trees, which is similar to the research of [28], which uses Multi Layer Perceptrons (MLP) for detecting service level agreement violations. One large research topic is also the prediction of the next events during runtime, for which graph theory [25], LSTMs [29], Decision Trees [30–32], Markov Classifiers [31–33] or Multi Layer Perceptrons [34] are used.

With the works of [35, 36] there has been research in the process learning domain recently, which allows learning on complex symbolic sequences by combining various of their contained properties. As their proposed feature representations and general systems differ from our envisioned approach in crucial ways (e.g. by not numerically encoding the temporal information, or not covering multi-class classification problems), a direct application of and comparison with these systems is out of the scope of this publication. In [36] the authors are using LSTM to simultaneously allow the runtime prediction of the next events and the prediction of the remaining time to case completion. For their feature representation they combine different temporal properties of their data (relative timestamp, time within the day and within the week). While the concrete time features would be irrelevant or even misleading in our use case, the relative timestamp are very similar to our *t*_*e*.*rel*_. In [35] the authors are using hidden Markov Models and Decision Trees for predicting the achievement of a performance objective, as well as the fulfillment of a compliance rule. While being more complex, the features used in this work are only structured-sequential, i.e. the temporal component is not included in a quantitative form, thus missing an important requirement for our use case. Besides those differences both approaches do not allow for a multi-class prediction based on manually assigned class labels, as required in our use case. The highly dynamic, often non-deterministic behavior of the mobile network communication log sequences analyzed here, as well as their large number of states and transitions further hinders a direct application of these approaches. Those reasons also prevent the direct application of methods proposed in recent research in deep neural networks and their application on graph data [37–39], which opened novel ways of learning on complex data, e.g. for semi-supervised learning approaches or implicit feature spaces. Additionally, practical necessities like system interpretability and reliability are harder to fulfill with these more complex learning methods—as are the higher requirements on the data set sizes, necessary to achieve converging models of the trained neural networks.

Since we are operating on sequential data, one could also use methods originally from the field of *sequence labeling*, where the task is to predict a label for each event within the sequence, which was solved using methods like Hidden Markov Models [40], Conditional Random Fields [41], MLP [42], but recently also Recurrent Neural Networks and specifically LSTM Neural Networks [43–45]. Since in our objective data each event is already labeled though, we are not interested in predicting such individual labels, but instead require predictions based on the behavior represented by the complete sequences. Using predictions of such individual labels to describe a complete sequence label is also no option, as a defined sequence label can depend on structural-temporal properties not represented in event-wise predictions. Since the event order and the inter-event durations are semantically relevant, using methods of sequence alignment [46] is also not directly possible. Also methods like dynamic time warping [47] are not directly applicable, as they are not designed to process temporal data with additional structural properties, or multi-client dependencies. As such we need methods producing a label for the whole sequence, as is done in *sequence classification* [48, 49]. Of those methods *support vector machines* [50–56] are a well established method for *feature-based* sequence classification, which we are specifically interested in as we are trying to reflect the data properties by specific feature spaces. SVM approaches have shown a reliable performance, specifically when using non-sequential *n*-gram feature spaces for the respective sequential data, as done in the fields of natural language processing [13–16], intrusion detection [17, 18], malware detection [57–59] and the analysis of network communication [19–21, 60].

All of this shows that our approach to multi-class detection and prediction of network communication sequence failures can be distinguished from other approaches of related work and state-of-the-art methods, extending their concepts by the explicit integration of temporal properties and positional variance into the applicable feature space, as well as the inclusion of calibratable multi-class prediction compatibility. To achieve an evaluation which allows a comparison with these works, some of their most commonly used methods are used for conducting the experiments, as described in Subsection Learning Methods. While some approaches and features are in principle similar, we have refrained from applying our approach to standard problems of sequence learning or process mining, as this interesting line of research would deserve a publication of its own right and focus. Instead to remain focussed, we have compared only some of the process mining methods and features that are indeed applicable to the network communication application of this work.

## Use case description

We will now discuss the properties of the data and the contained problem classes of our concrete use case of failure classification for sequence data of mobile communication, and why these are representative for the analysis of structural and temporal aspects.

### Data set properties

We are using mobile communication data of a specific format as a concrete example to discuss and show the properties of sequential structural and temporal data. It was recorded to provide a wide-ranging quality analysis of the underlying network infrastructure. The data is collected by a fleet of specialized cars equipped with roof-mounted antennas and multiple android smartphones. The mobile phones run test sequences, which consist of automatically calling a phone within one of the other cars, establishing a connection, playing a voice chat of 1 minute and finally closing the connection. This whole process is monitored, recorded and consecutively analyzed by a system which focuses on various key performance indicators (KPIs) and statistics, radio frequency (RF) values and the successful completion of the key processing steps of the respective protocol state machines of UMTS, LTE and GSM. Since the cars are moving while all of this takes place, the recorded data also contains switches between different transmission technologies (e.g. the sequence may start in GSM, switches then to LTE, and finishes in UMTS). The resulting data already allows for drawing simple conclusions, e.g. on whether communication sequences have been successful at all (i.e. the relevant KPI and RF values did not show negative deviations, and all relevant protocol states have been completed successfully), whether they dropped in the middle (i.e. important protocol states have not been completed), or whether they have failed for other reasons.

Those failed sequences are then manually analyzed to determine their reason of failure and potentially even its cause. This process is called *failure classification*, allowing to assign a specific failure class to a failed sequence. Doing this manually by looking at many hundred log file entries is a tedious, time-consuming and expensive task. By using statistics over the KPIs, RF values and the protocol states for rule-based approaches, this can partially be automated, specifically for failures with simpler behavioral patterns. A more versatile machine learning approach could however help solving this problem better, potentially allowing to cover less clearly defined failure classes, while also adding some flexibility when trying to find new failure classes, caused by changes in the communication technology backbone architecture, e.g. with the upcoming 5G [61] technology.

For our analyses we collected two different data sets: the MFC data set, containing manually labeled and unlabeled failure class samples, and the AFC data set, containing failure class samples which are automatically labeled by a rule-based approach, as well as unlabeled samples. Table 1 shows some of their most important characteristics. Each contained sample represents a call sequence, which utilizes at least the GSM, UMTS or LTE protocol, following its respective specification and call phases, which is reflected in the logged events and the respective timestamps. Instead of using all the recorded events though, we are mainly interested in those that are relevant for the potential failure classes. Hence we are using filters based on rules that have been defined by experts with deep domain knowledge, removing all events that contain redundant or irrelevant information. As a result we obtain a final set of base events, which together with their different states (e.g. different reasons for a location update reject), and in combination with the respectively utilized protocol, leads to overall 335 different event identifiers.

**Table 1:** Data set statistics: Number of events *e*_#_, of failure sub classes *c*_#_ and of samples *s*_#_.

	MFC		AFC	
Average *e*_#_	44.11 ± 13.51		29.75 ± 27.24	
Main failure classes	*c*_#_	*s*_#_	*c*_#_	*s*_#_
CSFB Failure	2	48	2	370
Congestion Failure	-	-	1	60
Core Network Failure	1	30	8	682
E2E Failure	1	19	2	169
UE Failure	1	21	-	-
Other Failures	39	86	39	360
Sum	44	204	52	1,641
Unlabeled Samples	-	5,873	-	1,623

Both data sets will serve different purposes in this paper, because the details of the different labeling approaches used for both data sets are expected to impact the AFC evaluation performance negatively. The filtered events available in both data sets are selected to cover all important call phases and event sequences potentially relevant for a proper class prediction. As such they are theoretically sufficient to properly reproduce the MFC labels. However, they are insufficient to achieve a similar performance for the AFC data, where also data of additional events, as well as relevant KPI and RF data has been used for defining the classes, none of which we have access to in our structural-temporal data. As a result of these restrictions Table 1 shows that the average number of events per sequence is much smaller for AFC data, and its variance is much larger, reflecting a larger class variance and making a proper discrimination harder. Additionally, the AFC data contains 8 very similar variations of the *Core Network Failure* class, which are harder to discriminate as well. Due to these shortcomings of the AFC data set, the smaller MFC data set is more relevant for our purpose, as its labels provide a better ground truth. Since this is a problem in the AFC data set, we will restrict our analyses on the AFC data to those which are expected to provide valuable insights on this data set only, namely how well the proposed feature spaces and learning methods can still reproduce the AFC labels under these conditions, and—given its larger number of samples—how much of a performance improvement we can expect when increasing the training data size.

We will now discuss the format of the contained sequences. Each sample in our data sets consists of the bi-directional communication sequence between a caller and a callee. We denote the caller as the MOC client (mobile originated call) and the callee as the MTC client (mobile terminated call). The samples contain highly structured sequential data (order of events) with highly relevant temporal components (inter-event durations), all of which are semantically relevant for discriminating the failure classes, e.g. reflecting call phases being incomplete or too long, or reflecting an anomalous order of events. Table 2 shows an exemplary event sequence of a successful call, starting in LTE and proceeding and ending in UMTS. This example also introduces the new timestamp format *t*_*s*.*rel*_, denoting sequence-relative timestamps, defined for a sequence *s* as *t*_*s*.*rel*_(*e*_*i*_) = *t*(*e*_*i*_) − *t*(*e*_0_) (or *t*_*s*.*rel*_(*s*)_*i*_ = *t*(*s*)_*i*_ − *t*(*s*)_0_), i.e. the timestamp of the first sequence event is set to 0.0, and all other timestamps are reset relative to this value. In the example the MOC client is set up until *t*_*s*.*rel*_ = 12.232, then the MTC client is set up until *t*_*s*.*rel*_ = 14.231. Once the clients are connected at *t*_*s*.*rel*_ = 30.103 the call takes place, before they are finally disconnected again. The abbreviations represent the following event types: extended service (ES) request, security mode (SM) command and complete, connection management service (CMS) request, radio bearer (RB) setup and extended service (ES) request.

**Table 2:** Example sequence of successful bi-directional mobile communication.

*t*_*s*.*rel*_	MOC client		MTC client	
	protocol	event	event	protocol
0.0	LTE	ES Request		
1.271	LTE	SM Command		
1.271	LTE	SM Complete		
2.385	UMTS	SM Command		
2.386	UMTS	SM Complete		
3.298	UMTS	CMS Request		
3.864	UMTS	SM Command		
3.864	UMTS	SM Complete		
4.273	UMTS	Setup		
4.826	UMTS	Call Proceeding		
5.192	UMTS	RB Setup		
5.392			ES Request	LTE
6.209			SM Command	LTE
6.209			SM Complete	LTE
12.232	UMTS	RB Setup Complete		
	MOC client is set up			
12.577			SM Command	UMTS
12.577			SM Complete	UMTS
13.130			Setup	UMTS
13.253			Call Confirmed	UMTS
13.783			RB Setup	UMTS
14.231			RB Setup Complete	UMTS
			MTC client is set up	
14.799			Alerting	UMTS
14.865	UMTS	Alerting		
15.721			Connect	UMTS
16.254			Connect Ack	UMTS
18.237	UMTS	Connect		
19.020	UMTS	Connect Ack		
29.923	UMTS	SM Command		
29.924	UMTS	SM Complete		
30.032	UMTS	RB Setup		
30.103	UMTS	RB Setup Complete		
	Clients are connected and the call takes place			
93.028	UMTS	Disconnect		
93.427			Disconnect	UMTS

### Failure classes

Before analyzing how the concrete sequence properties are utilized in the feature spaces, we first need to discuss the existing failure classes and their structural and temporal properties in more detail, to provide a better practical background of the problem domain. Table 1 contains an overview and provides relevant class statistics for the selected, sufficiently sized failure sub-classes of the listed main failure classes. It also contains details about the samples of insufficiently sized failure classes, grouped together in the set of *Other Failures*, as well as the additional number of unlabeled samples per data set.

#### CSFB problems

A circuit switch fallback is conducted when the current network (e.g. LTE) does not sufficiently fulfill the current connection requirements (e.g. signal strength, cell coverage, sufficient response times) or suffers from other problems, while at the same time an older network (e.g. GSM) is available. It can also occur when one of the communicating clients is not LTE capable. Handing over the correct connection state to another protocol can be problematic, though. Our data set contains cases of failures that occurred when the call setup was not properly continued after the location area update (LAU) and the routing area update (RAU), when the current network did not allow a proper release for redirection, or when the redirection to the older network simply took too long.

#### Congestion problems

These problems can occur when the network is overloaded, s.t. problems with the connection or response timings occur. In our data sets we have sample sequences where the connection downlink disconnected too early, leading to an interrupted connection, and other cases, where no circuit channel was available, completely preventing to establish a connection.

#### Core network problems

This failure class represents more general problems, where the causes might be similar to those of the previous failure classes, e.g. an unexpected downlink disconnect or problems with LAU and RAU. The previous classes, however, contain additional semantic properties that lead to the classification as CSFB (fallback to older technology) or congestion problem (high latency, low bandwidth), which are missing here. Failure sub classes dominant in our data sets contain cases of unexpected downlink disconnect, unreachable MTC clients, or no or too slow reply to LAU or RAU. The high similarity to other classes, as well as the fact that only minor differences exist between its individual sub classes makes discriminating them harder, which is specifically relevant for the AFC data, with its higher number of samples of these sub-classes.

#### E2E problems

End to end problems occur beyond the scope of the core network. In our data set two of its sub classes are prominently represented. Unexpected downlink (DL) radio resource control (RRC) connection releases are symptoms of problems during the downlink authentication phase of the connection, causing it to fail. This also holds for missing downlink setup failures, which already fail at an even earlier state.

#### UE problems

Besides those network and protocol related failures, problems can also occur on the devices themselves. The MFC data set contains samples of potential firmware issues, leading to such problems.

#### Other failures

Sequences of failure classes that contain only very few samples are not useable for a proper evaluation. Those samples are all re-labeled as *Other Failures*, allowing to use them in the model class detection step of our system.

## Structural and temporal feature spaces

One of the objectives of this paper is to analyze feature spaces capable of representing sequential data with structural and temporal properties, like the one detailed in the previous section, and to propose a feature space suited to better represent those properties. To achieve this, we discuss five different feature spaces Γ_*qT*_, Γ_*T*_, Γ_*S*_, Γ_*S*+*T*_ and Γ_*ST*_. Γ_*qT*_ is based on a sequential representation of the data, as commonly used in process mining [23]. Since we are specifically interested to additionally integrated temporal information in our feature spaces, Γ_*qT*_ optionally allows the inclusion of quantized temporal information. Γ_*T*_ focusses on the temporal information in a re-ordered sequential representation, while Γ_*S*_ focusses on the structural information in a non-sequential representation, essentially using a token n-gram approach, as described in Section Related Work. Finally we propose Γ_*S*+*T*_ and Γ_*ST*_ to show the advantages of integrating structural and temporal information complementarily into a single feature space, which is expected to allow a better data representation, compared to using only structural or temporal features alone. We discuss those feature spaces abstract, but also discuss unique properties that are specifically relevant for our use case. As such some of those discussions are exemplary adaptations of the abstract feature space properties to our concrete use case. However, this should not be seen as a restriction on the general applicability of these feature spaces, as they can be adapted to other use cases as well.

### Base processing

All of the following feature spaces require an identical base processing, for which we need a second timestamp format, enabling the consideration of relative time-dependencies (i.e. local delays): the event-relative timestamps *t*_*e*.*rel*_, defined as *t*_*e*.*rel*_(*e*_*i*_) = *t*_*s*.*rel*_(*e*_*i*_) − *t*_*s*.*rel*_(*e*_*i*−1_) (or *t*_*e*.*rel*_(*s*)_*i*_ = *t*_*s*.*rel*_(*s*)_*i*_ − *t*_*s*.*rel*_(*s*)_*i*−1_) for a given sequence *s*.

One objective for our proposed feature spaces is, that they represent multi-client behavior within the sequential data. This is relevant for our use case, because some failures can be caused by erroneous behavior on the MOC side of the call, while others are caused by problems on the MTC side—or even by problems on both sides, individually or combined. To reflect those structural properties, we need to create the sub-sequences *s*_*MOC*_ and *s*_*MTC*_ to contain exclusively the events of MOC and MTC respectively, with |*s*_2*C*_| = |*s*_*MOC*_| + |*s*_*MTC*_|. These representations allow the feature spaces to omit events of the respective opposite client. Table 2 shows this behavior exemplarily for the MOC-events at *t*_*s*.*rel*_ = 5.192 and *t*_*s*.*rel*_ = 12.232. They are interrupted by three MTC events in the *s*_2*C*_ sequence, but are represented consecutively in the *s*_*MOC*_ sequence. As a result, a sequence *s* can be described by a triple of sequences *s*_2*C*_, *s*_*MOC*_ and *s*_*MTC*_. If not stated otherwise, we use *s* synonymously with *s*_2*C*_ to denote the complete 2-client communication. As such the example in Table 2 effectively illustrates an *s*_2*C*_ sequence. This definition is extended to the whole set of sequences. Where previously we denoted all sequences *s* in a set *S* as *s* ∈ *S*, we can now also denote the sets of sequences of each different representation, i.e. *s*_2*C*_ ∈ *S*_2*C*_, *s*_*MOC*_ ∈ *S*_*MOC*_ and *s*_*MTC*_ ∈ *S*_*MTC*_. This also allows extending the definition of *t*_*s*.*rel*_ to those representations, in that *t*_*s*.*rel*_(*s*_2*C*_) denotes the vector of the sequence-relative timestamps of *s*_2*C*_, and *t*_*s*.*rel*_(*s*_*MOC*_) and *t*_*s*.*rel*_(*s*_*MTC*_) denote those of the client-wise sequence representations. The same holds for *t*_*e*.*rel*_. Note that *t*_*s*.*rel*_ is set to 0.0 for the first event of each sequence representation respectively (i.e. *t*_*s*.*rel*_(*s*)_*i*_ = *t*(*s*)_*i*_ − *t*(*s*)_0_ for *s* ∈ {*s*_*MOC*_, *s*_*MTC*_}), to allow for a better comparability of sequences of the same client type.

Using an exemplary set of event identifiers *E* = {′*A*′, ′*B*′, ′*C*′, ′*D*′} allows creating two artificial example sequences *x*_1_ and *x*_2_ and their timestamps in the two formats, as shown in Table 3. These will be used in the next sections to illustrate various aspects of the different feature spaces.

**Table 3:** Two artificial communication sequences *x*_1_ and *x*_2_.

*x*_1_			*x*_2_		
*t*_*s*.*rel*_	*t*_*e*.*rel*_	*e*_*i*_	*t*_*s*.*rel*_	*t*_*e*.*rel*_	*e*_*i*_
0.0	0.0	D	0.0	0.0	D
2.211	2.211	A	1.823	1.823	B
4.341	2.130	B	3.230	1.407	B
6.207	1.866	C	5.103	1.873	C
18.075	11.868	A	25.330	20.227	B
			30.548	5.218	A

### Γ_*qT*_ features

Γ_*qT*_ features are based on the *s* ∈ *S*_2*C*_ feature vectors, essentially representing the most common type of data representation used in the related work of process mining. We extend this representation additionally by quantized temporal features. The idea is to get a sequential representation of the event identifiers, which is specifically suited for classifiers used in process mining, but to additionally include temporal information. To achieve this, we start with the event indices in *s*. Consecutive events with a temporal distance closer than a predefined minimum interval *θ*_*mi*_ maintain their current position in the event index sequence. If consecutive events exceed *θ*_*mi*_, an additional empty event *e*_∅_ is inserted, reflecting the larger interval between those consecutive events. This is repeated until the next event is reached. As a result, smaller values of *θ*_*mi*_ introduce more events *e*_∅_ and lead to larger, sparser feature vectors, while larger values of *θ*_*mi*_ introduce less empty events, thereby decreasing the feature vector length while increasing the density—until a completely dense feature vector is achieved, containing no empty events *e*_∅_ at all. Through manual analysis of the temporal properties of the analyzed data, a value of *θ*_*mi*_ = 5.0*s* is selected as a compromise capable of filling large temporal gaps occurring in the data (which often represent overly long durations between two protocol states) while not increasing the overall feature vector length too much in less relevant regions of the sequence. For *θ*_*mi*_ = 5.0*s*, the resulting Γ_*qT*_ feature vector for sequence *x*_1_ in Table 3 is thus Γ_*qT*_(*x*_1_) = [′*D*′, ′*A*′, ′*B*′, ′*C*′, *e*_∅_, *e*_∅_, ′*A*′], s.t. the large *t*_*e*.*rel*_(′*A*′) is represented by two instances of *e*_∅_. Choosing *θ*_*mi*_ > 60.0*s* allows eliminating all occurrences of *e*_∅_, which is identical to the original sequence of events, without the additional temporal information provided by the *e*_∅_ inserted in the sequence. When using Γ_*qT*_ in this way, we denote it as Γ_qT*_, which allows highlighting the performance differences when using both types of feature representations with competing classifiers of the process mining domain.

### Γ_*T*_ features

To create the set of *t*emporal features Γ_*T*_, we use the set *S*_2*C*_. The idea of Γ_*T*_ is to create a feature space which projects properties of the *i*th occurrence of each event type in a sequence *s* onto the same dimension. The projected property is the timestamp *t*_*s*.*rel*_ of the respective event, allowing to compare it with the timestamps of the *i*th occurrence of the same event type of other sequences. We start by calculating the occurrence frequency *f*(*e*, *s*) for each event type *e* ∈ *E* in each sequence *s* ∈ *S*_2*C*_. Then we define its maximum value as *m*_*e*_ = *max*(*f*(*e*, *s*)), ∀*e* ∈ *E*, ∀*s* ∈ *S*_2*C*_, calculating what is the most frequent occurrence of event type *e* in any sequence. Furthermore a function *κ*(*e*, *s*, *i*) is required, returning the *i*th occurrence of *e* in *s*. For example the simple sequence *x*_1_ in Table 3 has two occurrences of the event type ‘A’. As such, *κ*(′*A*′, *x*_1_, 2) returns *e*_5_ = ′*A*′, i.e. the 5th event in *s* is the 2nd occurrence of ‘A’ in *s*, with *t*_*s*.*rel*_(*κ*(′*A*′, *x*_1_, 2)) = 18.075. Now a function *ts*(*s*, *e*, *m*_*e*_) can be defined, providing a vector of these timestamps *t*_*s*.*rel*_ of all occurrences of a selected event in a sequence, and 0.0 otherwise:

$$\begin{array}{c} ts\left(s,e,m_{e}\right)=\left[\tau \left(s,e,1\right),\ldots ,\tau \left(s,e,m_{e}\right)\right] \end{array}$$

with

$$\begin{array}{c} \tau \left(s,e,i\right)=\{\begin{array}{cc} t_{s.rel}\left(\kappa \left(e,s,i\right)\right) & \text{if}\;i\leq f(e,s)\\ 0.0 & \text{else} \end{array} \end{array}$$

Concatenating the resulting vectors of *ts*(*s*, *e*, *m*_*e*_) for a sorted list of all *e* ∈ *E* via the concatenate()-function yields the final feature vector for a sequence *s*, which has for all samples the same length of ∑*m*_*e*_, ∀*e* ∈ *E*. A small example should make this better understandable. Table 3 shows two simple sequences {*x*_1_, *x*_2_} = *X*, for which the maximum frequencies *m*_*e*_ = *max*(*f*(*e*, *x*)), ∀*x* ∈ *X* of each *e* ∈ {′*A*′, ′*B*′, ′*C*′, ′*D*′} are *m*_*A*_ = 2, *m*_*B*_ = 3, *m*_*C*_ = 1, *m*_*D*_ = 1. As a result the feature vectors have the following format:

$$\Gamma_{T}\left(s\right)=\text{concatenate}\left(\left[ts\left(s{,}^{\prime }A^{\prime },2\right),ts\left(s{,}^{\prime }B^{\prime },3\right),ts\left(s{,}^{\prime }C^{\prime },1\right),ts\left(s{,}^{\prime }D^{\prime },1\right)\right]\right),$$

for *s* ∈ {*x*_1_, *x*_2_}, resulting in the following final feature vectors:

$$\begin{array}{c} \begin{array}{cc} \Gamma_{T}\left(x_{1}\right) & =\text{concatenate}(\left[ts\left(x_{1}{,}^{\prime }A^{\prime },2\right),ts\left(x_{1}{,}^{\prime }B^{\prime },3\right),ts\left(x_{1}{,}^{\prime }C^{\prime },1\right),ts\left(x_{1}{,}^{\prime }D^{\prime },1\right)\right])\\ & =\text{concatenate}([[2.211,18.075],[4.341,0.0,0.0],[6.207],[0.0]])\\ & =[2.211,18.075,4.341,0.0,0.0,6.207,0.0] \end{array} \end{array}$$

and

$$\begin{array}{c} \begin{array}{cc} \Gamma_{T}\left(x_{2}\right) & =\text{concatenate}(\left[ts\left(x_{2}{,}^{\prime }A^{\prime },2\right),ts\left(x_{2}{,}^{\prime }B^{\prime },3\right),ts\left(x_{2}{,}^{\prime }C^{\prime },1\right),ts\left(x_{2}{,}^{\prime }D^{\prime },1\right)\right])\\ & =\text{concatenate}([[30.548,0.0],[1.823,3.230,25.330],[5.103],[0.0]])\\ & =[30.548,0.0,1.823,3.230,25.330,5.103,0.0] \end{array} \end{array}$$

To obtain an optional binary representation of Γ_*T*_, values of *τ*(*s*, *e*, *i*) larger than a defined threshold can be set to 1, and to 0 otherwise.

### Γ_*S*_ features

The structural Γ_*S*_ features are event *n*-gram features, similar to the previously used token *n*-gram features, and as such representing a feature representation commonly used in the related works of sequence classification. Therefore we extract for all *s*_2*C*_, *s*_*MOC*_ and *s*_*MTC*_ all event *n*-grams and index their sorted list, spanning the final feature space Γ_*S*_—including the client-specific *n*-grams of *s*_*MOC*_ and *s*_*MTC*_. We denote an event *n*-gram of a sequence feature vector *s* via its vector indices in interval notation (similar to the one used in Matlab or numpy), s.t. *s*_[*i*,*i*+*n*)_ denotes the event *n*-gram from position *i* (inclusive) to position *i* + *n* (exclusive). The Γ_*S*_ feature vector of sequence sample *s* is then defined via the binary occurrence of the respective event *n*-gram within *s*. When using the examples *x*_1_ and *x*_2_ from Table 3, the sorted list of *n*-grams for *n* = 3 is [‘DAB’, ‘ABC’, ‘BCA’, ‘DBB’, ‘BBC’, ‘BCB’, ‘CBA’], resulting in the final Γ_*S*_ feature vector Γ_*S*_(*x*_1_) = [1, 1, 1, 0, 0, 0, 0].

### Γ_*S*+*T*_ features

One base hypothesis of this paper is that the classification performance can be increased by using a complementary structural and temporal feature space. For the structural-temporal Γ_*S*+*T*_ feature space we treat the feature vectors of Γ_*S*_ and Γ_*T*_ as equivalent. Because of its binary format, Γ_*S*_ already produces qualitative feature vectors, but Γ_*T*_ produces quantitative feature vectors. If we binarize its values, we create a qualitative representation, which we can simply concatenate with the Γ_*S*_ feature vector. This is used here to provide a baseline complementary feature space, before defining the more complex complementary feature space Γ_*ST*_.

### Γ_*ST*_ features

For the structural-temporal Γ_*ST*_ feature space we will first need an analysis of the representative capabilities we specifically want to achieve with this feature space. As such, we will start this section with an analysis of some feature requirements, before explaining how these requirements are met by creating the data representation via structural-temporal *δ* − *n* matching and the use of model sequences.

#### Context and position

Metrics and features for structured, sequential data should reflect its specific properties. A sample of such data could be described by the occurrence of single *n*-grams (as done in Γ_*S*_). But this description can be improved when these *n*-grams are also analyzed in terms of their broader context and position. As such two similarly positioned *n*-grams might be identical, but their respective neighbor events (their context) might be different, which should prevent or penalize a match between them. This is highly relevant in data which is created by protocol-driven processes, like mobile communication data, which follows specific protocol states (e.g. for the radio bearer setup or the security parameter negotiations), all requiring specific events in their context. Thus it is important to focus on comparing contextualized *n*-grams with each other, i.e. events at the call setup should not be compared with those in the final call phases.

#### Model sequences

For the definition of Γ_*ST*_ the concept of *model sequences* needs to be introduced. Projecting each of the *s* ∈ *S* onto a feature space spanned by these model sequences yields projected samples of the same length (independent from the length of the projected sequence *s*), while at the same time incorporating both temporal and structural properties. The use of model sequences is based on the idea of defining the features of a sequence *s* based on its similarity to each model sequence *s*^*M*^ in the set of model sequences *S*^*M*^, which thus defines a feature space *model*. To this purpose we define the set of model sequence representations as a triple $S^{M}=\left(S_{2C}^{M},S_{MOC}^{M},S_{MTC}^{M}\right)$ just as we did for our actual sequences. By consecutively indexing the sequences and the events within *S*^*M*^ we effectively span a feature space of size $\sum_{\forall s^{M}\in S^{M}}|s^{M}|$. Note that the model sequences do not have to be labeled, and also do not have be mutually exclusive to the set of training or test sequences *S* = {*S*_2*C*_, *S*_*MOC*_, *S*_*MTC*_}, as we are not using the labels of the model sequences in any way. We rely instead on the relevance of their contained structural and temporal properties, offering insight into relevant types of behavior, required for the class discrimination. However, as the feature space is spanned by using the model sequences, their labels could potentially be used to increase the contained number of different features, or to balance the representation of features of more complex failure classes against those of simpler ones.

#### Defining the structural temporal features

The Γ_*ST*_ feature space is based on the idea of representing structural and temporal properties of the respective sequences. In this paragraph we will discuss, how to achieve this by using *n*-grams and model sequences in structural-temporal matching procedure, with a focus on the feature properties of context and position. We define the context of each event by the size of the *n*-grams and the parameter of positional variance *δ*, and the positional properties by the actual matching procedure. The idea of this procedure is to match each *n*-gram of each *s* ∈ *S* with the *n*-grams of each *s*^*M*^ ∈ sorted(*S*^*M*^), requiring identical positions of both matching *n*-grams within the two sequences—and then loosen this requirement via the parameter *δ*. The result of this matching for each *s* ∈ *S* is a vector of its structural similarity to each of the model sequences *s*^*M*^, for each of its sequence representations *s*_2*C*_, *s*_*MOC*_ and *s*_*MTC*_. Specifically the additional matches on the $S_{MOC}^{M}$ and $S_{MTC}^{M}$ are relevant for failures, as they allow detecting event chains of a single client, automatically crossing the gap caused by interfering events of the other client. We achieve this by a structural *δ*-*n* matching function, denoted as $\hat{\Phi }\left(s,s^{M},{\hat{s}}^{M},\delta ,n\right)$, where ${\hat{s}}^{M}$ denotes a vector of length |*s*^*M*^|, which is initialized as a zero-vector. By iterating over all indices, this vector is populated as follows:

$$\begin{array}{c} \hat{\Phi }\left(s,s^{M},{\hat{s}}^{M},\delta ,n\right)=\{\begin{array}{cc} \text{inc}({\hat{s}}_{\left[i+j,i+n+j\right)}^{M},{\vec{1}}_{n}) & \text{if}\;s_{\left[i+j,i+n+j\right)}^{M}=s_{\left[i,i+n\right)}\\ {\hat{s}}_{\left[i+j,i+n+j\right)}^{M} & \text{else} \end{array} \end{array}$$

∀(*i* + *j*) ≥ 1 and ∀(*i* + *n* + *j*) ≤ |*s*^*M*^| + 1, with *i* ∈ [1, |*s*^*M*^| − *n* + 1], *j* ∈ [−*δ*, *δ*] and *δ* ≥ 0. $\text{inc}\left(\vec{x},\vec{y}\right)=\vec{x}+\vec{y}$ denotes here the element-wise incrementation of vector ${\hat{s}}^{M}$ by a one-vector $\vec{1}$ of length *n*. Here we are using the indexing method introduced for Γ_*S*_ to denote individual event *n*-grams, s.t. *s*_[*i*,*i*+*n*)_ denotes the event *n*-gram of the events [*e*_*i*_, …, *e*_*i*+*n*−1_] in the sequence *s*. As such we essentially compare *s*_[*i*,*i*+*n*)_ with $s_{\left[i+j,i+n+j\right)}^{M}$, and allow a positional variance in the model sequence by defining *j* over the range of [−*δ*, *δ*].

Using the exemplary sequence *x*_1_ and *x*_2_ of Table 3, with *x*_1_ as

$$\begin{array}{c} s={[}^{\prime }D^{\prime }{,}^{\prime }A^{\prime }{,}^{\prime }B^{\prime }{,}^{\prime }C^{\prime }{,}^{\prime }A^{\prime }] \end{array}$$

and *x*_2_ as model sequence

$$\begin{array}{c} s^{M}={[}^{\prime }D^{\prime }{,}^{\prime }B^{\prime }{,}^{\prime }B^{\prime }{,}^{\prime }C^{\prime }{,}^{\prime }B^{\prime }{,}^{\prime }A^{\prime }], \end{array}$$

the structural *δ*-*n* matching with *δ* = 1 and *n* = 1 yields the following matching results for the respective values of *i* and *j*

**Table:** 

	*j* = −1	*j* = 0	*j* = 1	${\hat{s}}_{\left[i+j,i+n+j\right)}^{M}$					
*i* = 1	-	$s_{1}^{M}=s_{1}$	$s_{2}^{M}\neq s_{1}$	[1,	0]				
*i* = 2	$s_{1}^{M}\neq s_{2}$	$s_{2}^{M}\neq s_{2}$	$s_{3}^{M}\neq s_{2}$	[0,	0,	0]			
*i* = 3	$s_{2}^{M}=s_{3}$	$s_{3}^{M}=s_{3}$	$s_{4}^{M}\neq s_{3}$		[1,	1,	0]		
*i* = 4	$s_{3}^{M}\neq s_{4}$	$s_{4}^{M}=s_{4}$	$s_{5}^{M}\neq s_{4}$			[0,	1,	0]	
*i* = 5	$s_{4}^{M}\neq s_{5}$	$s_{5}^{M}\neq s_{5}$	$s_{6}^{M}=s_{5}$				[0,	0,	1]
			${\hat{s}}^{M}$ =	[1,	1,	1,	1,	0,	1]

resulting in the vector

$$\begin{array}{c} \hat{\Phi }\left(s,s^{M},{\hat{s}}^{M},1,1\right)=\left[1,1,1,1,0,1\right]. \end{array}$$

Since we are not only interested in the structural properties of our data, we will now extend $\hat{\Phi }$ by integrating the event-relative timestamps *t*_*e*.*rel*_ as temporal properties, obtaining the final structural-temporal projection function Φ. The idea is to calculate the absolute differences of the *t*_*e*.*rel*_ of the structurally matching events of *s* and *s*^*M*^. This is done by modifying the previously used incrementation function int(), giving rise to the final definition of Φ:

$$\begin{array}{c} \Phi \left(s,s^{M},{\hat{s}}^{M},\delta ,n\right)=\{\begin{array}{cc} \text{inc}({\hat{s}}_{\left[i+j,i+n+j\right)}^{M},\Delta_{abs}\left(\vec{x},\vec{y}\right)) & \text{if}\;s_{\left[i+j,i+n+j\right)}^{M}=s_{\left[i,i+n\right)}\\ \;\text{with}\;\vec{x}=t_{e.rel}{\left(s^{M}\right)}_{\left[i+j,i+n+j\right)}\\ \;\text{and}\;\;\vec{y}=t_{e.rel}{\left(s\right)}_{\left[i,i+n\right)}\\ {\hat{s}}_{\left[i+j,i+n+j\right)}^{M} & \text{else} \end{array} \end{array}$$

again ∀(*i* + *j*) ≥ 1 and ∀(*i* + *n* + *j*) ≤ |*s*^*M*^| + 1, with *i* ∈ [1, |*s*^*M*^| − *n* + 1], *j* ∈ [−*δ*, *δ*] and *δ* ≥ 0. The function $\Delta_{abs}\left(\vec{x},\vec{y}\right)$ defines a vector by calculating the absolute element-wise difference between two vectors $\vec{x}$ and $\vec{y}$, which is in this case the temporal difference of the respective matching events of *s* and *s*^*M*^, as illustrated in the previous example. As this can result in multiple matches per event in *ŝ*^*M*^, we finally average each field in *ŝ*^*M*^ by its number of matches. Applying this final formulation to the previous example sequences and their event-relative timestamps

$$\begin{array}{c} t_{e.rel}\left(s\right)=\left[0.0,2.211,2.130,1.866,11.868\right] \end{array}$$

and

$$\begin{array}{c} t_{e.rel}\left(s^{M}\right)=\left[0.0,1.823,1.407,1.873,20.227,5.218\right] \end{array}$$

yields the final feature vector

$$\begin{array}{c} \Phi \left(s,s^{M},{\hat{s}}^{M},1,1\right)=\left[0.0,0.307,0.723,0.007,0,6.65\right]. \end{array}$$

Since Φ is defined over a single *s*^*M*^, it has to be executed for all *s*^*M*^ ∈ sorted(*S*^*M*^), and the resulting vectors *ŝ*^*M*^ have to be concatenated via the concatenate()-function, giving rise to the final definition of Γ_*ST*_:

$$\begin{array}{c} \Gamma_{ST}\left(s,S^{M},\delta ,n\right)=\text{concatenate}\left(\Phi \left(s,s^{M},{\hat{s}}^{M},\delta ,n\right)\right),\forall s^{M}\in \text{sorted}\left(S^{M}\right) \end{array}$$

The final feature vector Γ_*ST*_(*s*, *S*^*M*^, *δ*, *n*) has the length $\sum_{\forall s^{M}\in s^{M}}|s^{M}|$, and contains the *structural-temporal δ* − *n matches* of the sequence *s* and all model sequences *S*^*M*^. The *O*-complexity of this whole process is linear, as creating the feature space by indexing the model sequences *S*^*M*^ is done in linear time, and projecting a single sequence *s* onto *S*_*M*_ is linear to the number of model sequences |*S*_*M*_|, as it requires matching the *n*-grams of each *s* with those of every *s*^*M*^ ∈ *S*^*M*^.

#### Explaining the semantics

The objective of our projection Φ is to achieve features highlighting differences in structurally similar, but temporally different sequences, i.e. we aim for a way to define similar features for sequences with similar structural and temporal behavior, while achieving different feature vectors for those which are structurally different, or which are structurally similar, but temporally different. As one can see, the value of a single dimension is 0 if there is no structural match, it is very small if *t*_*e*.*rel*_(*s*_[*i*,*i*+*n*)_) and $t_{e.rel}\left(s_{\left[i+j,i+n+j\right)}^{M}\right)$ are similar, and it is large if *t*_*e*.*rel*_(*s*_[*i*,*i*+*n*)_) and $t_{e.rel}\left(s_{\left[i+j,i+n+j\right)}^{M}\right)$ strongly deviate.

Once the feature vector of *s* is calculated, it is utilized in the classifier, where this feature projection does indeed allow focussing on the desired differences. If the projections of both samples have small values for a dimension, those small values contribute to a small distance between two samples, yielding a high similarity between the samples. If the projections of both samples have similarly large values for a dimension, these can contribute to a small distance between two samples—but only if they are similarly large. This is only the case if both samples have a similarly large deviation from the timestamps of the model sequence, which is only the case, if they show a similar temporal behavior. If the projections of both samples have differing values for a dimension, these values increase the distance between both samples, emphasizing their inter-sample difference for this dimension.

#### Further considerations

To improve the feature balancing in the final Γ_*ST*_, the sequences for *S*^*M*^ should be carefully selected. *S*^*M*^ containing an unbalanced number of samples per sequence class will lead to many, potentially redundant features for over represented data aspects (e.g. class specifics) or irrelevant properties (i.e. noise), while other data aspects could remain nearly uncovered, due to an insufficient number of *s*^*M*^ covering these. This makes multi-class learning harder, because these over represented features might outweigh less represented features and may therefore produce results prone to classify the corresponding class. While we made sure not to use duplicate sequences in any of our data sets, we did not include such an additional sequence selection optimization. To reduce the dimensionality of Γ_*ST*_, one should also select features that are most relevant for the classification task, e.g. by removing redundant features (e.g. those dimensions that are redundant between the single-client vectors *s*_*MOC*_ and *s*_*MTC*_, and the multi-client vector *s*_2*C*_), or by applying efficient feature selection methods like RDE [62].

Γ_*ST*_ also allows inspecting, which dimensions are of highest importance for the classification, allowing a knowledge transfer into potentially faster rule-based algorithms. Since the Γ_*ST*_ components also encode the temporal positions of the relevant *n*-grams, they can be mapped to additionally recorded radio frequency (RF) time-series data (e.g. reception level (RXLEV), reception quality (RXQUAL), received signal code power (RSCP)) of the logged communication sequences, enabling the detection of further RF-based failure classes like coverage or interference failures.

## System layout

**Fig 1:**
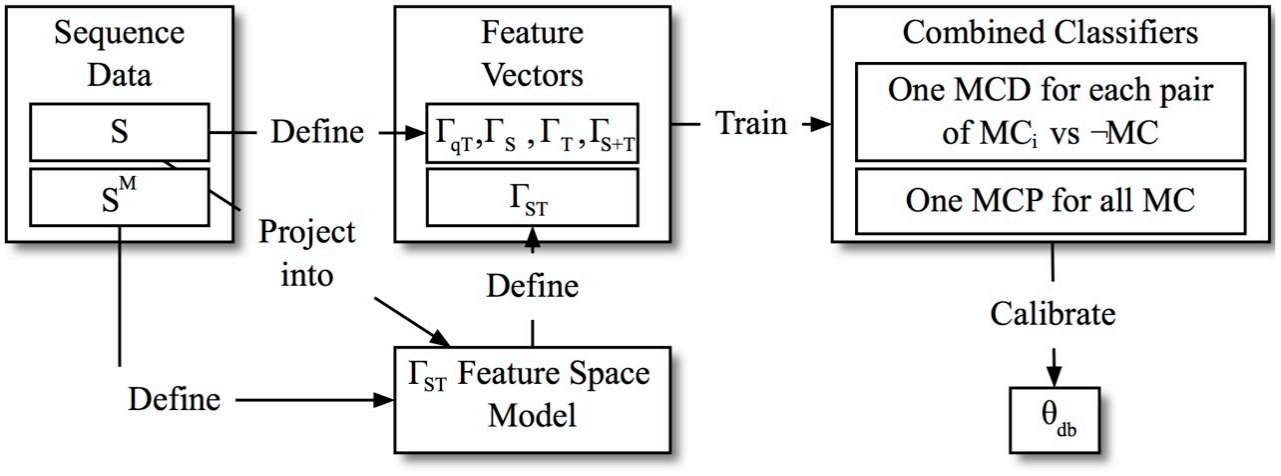
Training of the detection and prediction system.

This section will introduce the actual system for the detection and prediction of classes of sequence behavior. The system description will be kept as abstract as possible, to allow an application to other relevant use cases. Fig 1 shows the training phase of the system, in which the sets of sequences *S* are used to create the feature vectors, which are then used to train the required classifiers, responsible for the detection and prediction of properly represented model classes.

### Model class detection and prediction

In our use case, the classes of sequence behavior are defined by the different ways communication sequences between both clients can fail. When samples of failed sequences of a new data campaign need to be classified, we could assume a hypothetical scenario in which no new failure classes are found in the new campaign, i.e. all potential failure classes have already been seen before. However, this is not true in practice, where new types of previously unseen failures occur indeed. This is also true in our data sets, with the consequence that only a limited number of failures classes have a sufficient size to properly evaluate supervised classification *models* with them. We denote such classes as *model classes*, or *MC*. Sequences of *Other Failures*, i.e. of insufficiently large failure classes are denoted as *non model classes*, or ¬*MC*.

This motivates the design of our system, consisting of two major components, which allow to *detect* whether a new sample is potentially a *MC* sample (and not a sample of ¬*MC*), and if that is the case, to *predict* the respective model class. Accordingly, those steps are called the model class detection (MCD) and the model class prediction (MCP). For the MCP a classifier is trained in a multi class approach, learning to discriminate only samples of the *MC*, but not the ¬*MC*. For the MCD multiple classifiers are trained, one for each *MC*. Each of those classifiers is trained in a two class approach, learning to discriminate the respective *MC* against samples of ¬*MC*. After training the MCP and MCD classifiers, we can predict the failure class of a new sample by predicting a *MC* with the MCP classifier, and then using the MCD classifier trained for this *MC* to confirm or reject this prediction.

These predictions are obtained by applying their respective prediction functions to the feature vector Γ(*s*) of a test sample *s*, using one of the previously defined feature spaces, i.e. Γ ∈ {Γ_*qT*_, Γ_*T*_, Γ_*S*_, Γ_*S*+*T*_, Γ_*ST*_}. The function for the model class prediction classifier is *F*_*MCP*_, with

$$\begin{array}{c} y_{MCP}=F_{MCP}\left(\Gamma \left(s\right)\right) \end{array}$$

and with *y*_*MCP*_ ∈ {*MC*_1_, …, *MC*_*k*_}, the set of all *k* model class labels. The prediction function for the model class detector is *F*_*MCD*_(*MC*_*i*_), obtaining the prediction of the classifier trained for *MC*_*i*_ via

$$\begin{array}{c} y_{MCD}=F_{MCD}\left(MC_{i},\Gamma \left(s\right)\right) \end{array}$$

with *y*_*MCD*_ ∈ {*MC*_*i*_, ¬*MC*}. As one can see, *F*_*MCP*_ returns one of the *MC*-labels, and *F*_*MCD*_(*MC*_*i*_) returns the label of the class *MC*_*i*_, or ¬*MC*.

### Combined classification system

We are combining the prediction functions *F*_*MCP*_ and *F*_*MCD*_ by using two confidence ratings as a way to ensure a higher confidence in the predictions of the combined (MCP and MCD) classifier, as this helps improving the classification precision of our approach, which is crucial in the practical application. These confidence ratings produce the binary results *High* and *Low*. They can be logically combined and can be interpreted either as providing support for the prediction of the MCP (*High* confidence) or objecting against its prediction (*Low* confidence).

Since the output of *F*_*MCD*_ is limited to a single *MC*_*i*_ and ¬*MC*, it is used as the first confidence rating for *y*_*MCP*_, answering the question of whether sample *s* really belongs to the already predicted *y*_*MCP*_ (i.e. a specific *MC*_*i*_) or whether it belongs to ¬*MC*. For this purpose, the confidence rating function Θ_*MCD*_(Γ(*s*)) ∈ {*High*, *Low*} is defined as:

$$\begin{array}{c} \Theta_{MCD}\left(\Gamma \left(s\right)\right)=\{\begin{array}{cc} High & \text{if}\;F_{MCD}\left(y_{MCP},\Gamma \left(s\right)\right)=y_{MCP}\\ Low & \text{else} \end{array} \end{array}$$

To obtain further confidence on the classification result of *F*_*MCP*_, we define an additional confidence rating Θ_*db*_. It uses the decision boundary of the MCP classifier of the predicted class. The idea behind Θ_*db*_ is to calibrate the decision boundary of each MCP classifier towards more conservative values, requiring a sample with *y*_*MCP*_ = *MC*_*i*_ to cross a stricter decision boundary for this *MC*_*i*_ to obtain a *High* confidence for Θ_*db*_, which reduces the false positive rate and increases the precision. As it is defined over the decision scores, it can be applied to any classifier which provides access to its decision scores or probabilities. For this purpose we access the prediction score of the MCP via the function *D*_*MCP*_(Γ(*s*)). We also require the existing bias *b* of the MCP classifier of *y*_*MCP*_, and a parameter *θ*_*DB*_ ≥ 0 to define the new bias *b*_*db*_ = b + (*D*_⌀_ − *b*) · *θ*_*db*_, with *D*_⌀_ representing the mean of those decision scores of the training samples that have been correctly classified by the MCP. As such, the parameter *θ*_*db*_ gives rise to the following definition of the confidence rating Θ_*db*_:

$$\begin{array}{c} \Theta_{db}\left(\Gamma \left(s\right),\theta_{db}\right)=\{\begin{array}{cc} High & \text{if}\;D_{MCP}\left(\Gamma \left(s\right)\right)+b_{db}>0\\ Low & \text{else} \end{array} \end{array}$$

For example in the two-class definition of the SVM the decision function is *F*_*MCP*_(Γ(*s*)) = *sign*(*w*^*T*^Γ(*s*) + *b*), with *w* being the weight vector of the trained model. Here we achieve a positive prediction if *w*^*T*^Γ(*s*) + *b* > 0. The respective function to access the score is then *D*_*MCP*_(Γ(*s*)) = *w*^*T*^Γ(*s*) + *b*.

*θ*_*db*_ can be changed dynamically during model selection, which allows for a calibration of the model precision, similar to other methods of false positive calibration as used in e.g. [59]. The confidence ratings are then processed together with the prediction *y*_*MCP*_ to produce the final predicted label *F*_*combined*_(Γ(*s*)) ∈ {*MC*_1_, …, *MC*_*k*_, ¬*MC*}, defined as follows:

$$\begin{array}{c} F_{combined}\left(\Gamma \left(s\right)\right)=\{\begin{array}{cc} F_{MCP}\left(\Gamma \left(s\right)\right) & \text{if}\;\Theta_{MCD}\left(\Gamma \left(s\right)\right)\;=High\\ & \wedge \;\Theta_{db}\left(\Gamma \left(s\right),\theta_{db}\right)=High\\ \neg MC & \text{else} \end{array} \end{array}$$

**Fig 2:**
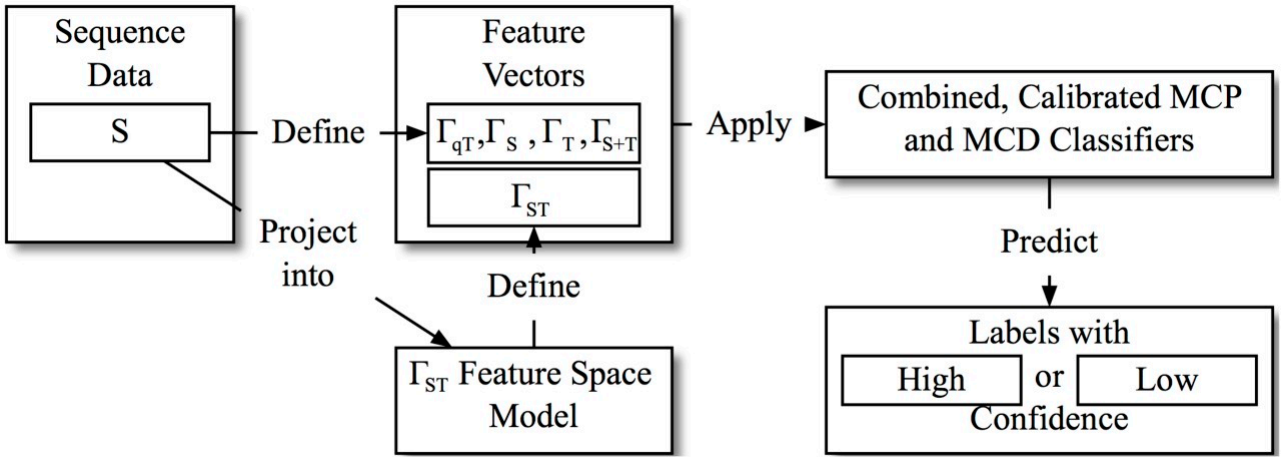
Application of the detection and prediction system.

The effect of the confidence ratings and the consequently created *High*-confidence prediction subset on the applied evaluation metrics will be elaborated in the next section. Now that the MCDs and MCPs are trained and calibrated, we can apply the system to classify unlabeled sequences, as illustrated in Fig 2. As just described, the final prediction *F*_*combined*_(Γ(*s*)) of this combined classifier for a given sequence *s* only provides the label of the predicted *MC*_*i*_ if Θ_*MCD*_(Γ(*s*)) = *High* and Θ_*db*_(Γ(*s*), *θ*_*db*_) = *High*, and ¬*MC* otherwise. This, together with the still accessible Θ-confidence ratings allows for an effective way of increasing the system classification precision, thereby discriminating reliable and unreliable predictions, which is highly relevant in the practical application.

## Evaluation

The evaluation section seeks to answer the following research questions:

- Which of the feature spaces achieve the best classification performance, when evaluated with suitable learning methods widely used in related work? Which learning method achieves the best and most robust results?
- Does the proposed Γ_*ST*_ feature space, which combines structural and temporal data properties in a novel way, achieve a better classification performance than other feature spaces, which do not use its additional properties of temporal features and positional variance?
- Under which circumstances does Γ_*ST*_ allow for a better classification performance than the baseline feature space Γ_*S*+*T*_? What are the implications for a practical application of the combined classification system?

To enable the analysis of these questions, subsection Learning Methods starts with describing the different learning methods used for the evaluation. To achieve a better reproducibility of the experiments, individual parameters and settings are described. Subsection Evaluation Metrics proceeds with explanations on the definition of the confusion matrix and additional metrics required in our use case to ensure the required practical applicability. The most important evaluations and analyses are then conducted in subsection Experiments on MFC data. This subsection starts with a description of the evaluation settings and the calibration of the *δ*-parameter, followed by the evaluation of the classification performances using various performance metrics for the individual MCP and MCD predictors, as well as their combined application to simulate the complete system workflow. The subsection ends with a statistical significance analysis, which further supports the achieved results. Finally subsection Experiments on AFC data describes evaluation results on the AFC data, thereby providing a different perspective on the proposed features and systems.

To enable reproducibility of the experiments, all data utilized in this manuscript is available in anonymized form as supporting material on the PLOS ONE publication page.

### Learning methods

While unsupervised or semi-supervised methods have shown to produce good results on textual and structural data and could be relevant to our problem of model class detection, supervised methods are regularly outperforming them and are the preferred solution, if labeled training data is available. As discussed in Section Related Work, Decision Trees, Markov Classifiers and LSTM are learning methods widely used in the domain of process mining, while MLP and SVM are more widely used on non-sequential data representations of sequential data. For these reasons we are conducting our evaluations on those methods, to provide a broad picture of the classification performances achievable on the discussed sequential (Γ_*qT*_), non-sequential (Γ_*T*_, Γ_*S*_, Γ_*S*+*T*_) and semi-sequential (Γ_*ST*_) feature representations. We also include the classification performance using k-nearest neighbors to provide an additional baseline. As some of the feature spaces are designed with specific learning methods in mind, they are only evaluated on those learning methods. In our experiments we actually tested all types of features with all learning methods, but achieved suboptimal results on non-suitable learning methods. For those reasons the respective results are omitted. All of the models below have been chosen using standard cross-validation based model selection.

#### Decision tree

Decision trees model training data based on their sequence, where their shared prefix paths build the root of the tree, which branches along the sequence down to the leafs, annotating the transitions with their respective probabilities. They are widely used, specifically in process mining in [27, 30–32] and as random forest of decision trees [35]. We use them as classifiers, based on the sequential Γ_*qT*_ features, using additional suffix padding to achieve equivalent length sequences.

#### Markov classifier

Markov Models are commonly used in process mining [24, 31–33, 63], where they are primarily used for predicting objectives like the remaining time or the next event, and not for sequence classification. It can also be used for classification though, as Markov Models represent the data of each class in the training data as a Markov process. This allows calculating the class-wise path probabilities for a new sequence, and predicting the most probable class by the highest overall path probability. We apply such a classifier on the sequential data representations of the Γ_*qT*_ feature spaces.

#### LSTM RNN

Recurrent Neural Network with Long Short-Term Memory nodes are a type of classifier recently used e.g. in process mining [29, 36]. Deep Learning approaches work best with large training data sets. In our use case, getting a large amount of labeled samples is not easy, thus a deep learning approach might not be the best way to address this problem. However, recurrent neural networks (RNN) with long short-term memory (LSTM) units have shown great performance on sequence prediction problems, s.t. evaluating their performance on this problem is still highly interesting. For our experiments we are using the tensorflow [64] implementation of RNN with LSTM. The Γ_*qT*_ features are specifically designed with an LSTM RNN in mind. To achieve samples of homogenous length per batch, we added empty events to the end of each sequence. Since the history of each event is of specific relevance in the event sequence handling in LSTMs, this suffix-padding is a good solution, as it allows to assure that the starting events are not empty. In our experiments we achieve the best results when using one-hot label encoding, a single hidden layer of 20 nodes, 300 epochs and a batch size of 10.

#### KNN

K-nearest neighbor classifiers are classical distance-based baseline classifiers from the field of natural language processing. We achieve the best results with a value of *k* = 5, using the euclidean distance while additionally weighing points by the inverse of their distance, s.t. closer points have a larger impact.

#### MLP

Multi Layer Perceptrons are a widely used type of neural network sequence classifier, e.g. in [28, 34]. We achieve the best results by using a single hidden layer with 80 nodes and the identity function as activation function.

#### SVM

Support Vector Machines are a supervised learning methods, training a maximal margin separating hyperplane between linearly separable class data. While this can also be extended to non-linearly separable class data, we are using a linear kernel, which has shown very good results given sufficiently high-dimensional data, and specifically for protocol-based communication data [19–21, 60]. For the MCP evaluation we are using a one-vs-rest (OVR) approach, as this includes calculating a separating hyperplane for each model class *MC*, which allows a confidence calibration to optimize the system precision, as explained in the next section.

### Evaluation metrics

The general system application of reliably detecting and predicting known amidst unknown sequence classes, and its concrete practical application in failure classification enforces a focus on two primary objectives: (1) to obtain reliable predictions (2) for as many *MC* samples as possible. Precision, recall and the F1 score capture those aspects. For the evaluation of the MCP and the MCD classifiers they are calculated for multiple cross validation repetitions, s.t. we chose to calculate their unweighted class mean (denoted with the keyword *macro*), because we already configured the sampling procedure to produce similarly sized model classes. Whenever the ¬*MC* class participated in the evaluation (in MCD and combined classifiers) we excluded it from the calculation of the classwise mean values, because our focus is on the correct detection of *MC* samples—and not on the correct detection of ¬*MC* samples. As the set of ¬*MC* samples is much larger than each individual *MC*, this would otherwise lead to overly optimistic evaluation results. The formula below shows this approach exemplarily for the *macro precision*, where the ¬*MC* are implicitly contained in the calculation of the precision of each *MC*, but are not used as a primary class:

$$\begin{array}{c} precision=\frac{\sum_{\forall i\in [1,\ldots ,k]}precision\left(MC_{i}\right)}{k} \end{array}$$

By combining MCP and MCD classifiers and applying confidence ratings, we effectively create a filter, allowing us to focus solely on the created *High*-confidence subset of the predictions, which is designed to contain only those samples and labels which truly are of a model class *MC* and are correctly classified as such, thereby fulfilling objectives (1) and (2). Due to this mixture of multi-class (MCP) and two-class (MCD) predictions, we also have to adapt the metrics in the utilized confusion matrix, as described in Table 4.

**Table 4:** Metrics of the confusion matrix.

Set *P* of samples in the *High*-confidence subset	
TP	*s* ∈ *MC* classified as the correct *MC*_*i*_
FP	*s* ∈ *MC* classified as an incorrect *MC*_*i*_
	*s* ∈ ¬*MC* incorrectly classified as a *MC*
Set *N* of samples in the *Low*-confidence subset	
FN	*s* ∈ *MC* classified as the correct *MC*_*i*_
TN	*s* ∈ *MC* classified as an incorrect *MC*_*i*_
	*s* ∈ ¬*MC* correctly classified as ¬*MC*

Based on these values precision and recall are defined as usual, with *precision* = *TP*/*P* and *recall* = *TP*/(*TP* + *FN*). However, to correctly address objective (2) we have to calculate an additional *effective recall* by considering all existing *MC* samples, not only those in the *High*-confidence subset. Therefore we define the effective recall as the recall of correctly predicted samples of *MC* over the sum of samples in all *MC*, i.e. $effective\;recall=TP/\sum s_{\#MC_{i}},\forall MC_{i}$. Together with the precision over the *High*-confidence subset, this metric allows for a conclusive analysis of the overall system classification performance, as provided by the combined classification processing.

### Experiments on MFC data

To be able to apply the selected learning methods, we have to assure sufficiently sized failure classes. To obtain any model classes at all, we sample only from classes with a minimum size of 15 sequences. To improve the interpretability of our classification results we opted for similarly sized failure classes, which we achieved by limiting the size of each failure class to a maximum of 25 sequences. As such, the number of samples per *MC* used for the evaluation, *s*_#*MC*_, is 15 < *s*_#*MC*_ ≤ 25. To simulate the complete system, we also need members of the *Other Failures* class, of which we used all 86 available sequences, i.e. *s*_#¬*MC*_ = 86. In the MCD evaluation this allows highlighting the *detection* purpose of the method, as the ratio of samples of *MC*_*i*_ to ¬*MC* is approximately 1: 4. In the combined evaluation the ratio of all *MC* samples to ¬*MC* samples is approximately 1: 1, which helps in the interpretation of the classification results. For defining the Γ_*ST*_ features we used all 6,077 labeled and unlabeled MFC samples as model sequences *S*^*M*^. As previously described this use of the unlabeled sequences helps to extract additional information about the behavior of the projected sequence. To further reduce the high dimensionality of the resulting Γ_*ST*_ feature space, we additionally applied a dimensionality reduction, which utilizes the redundancy between the *S*_2*C*_ and *S*_*MOC*_, and the *S*_2*C*_ and *S*_*MTC*_ feature vectors, once they are projected with the structural *δ* − *n* matching of $\hat{\phi }\left(s,s^{M},{\hat{s}}^{M},\delta ,n\right)$. This resulted in a feature space of 294,435 dimensions. We also conducted an additional analysis on the number of dimensions actually relevant for a sufficient data representation, which could motivate further dimensionality reduction steps. This analysis is summarized in the Appendix.

For all evaluations we used 20 times random sampling, each with a 5-fold cross validation. For a proper evaluation we made sure all conducted comparisons between classifiers and feature spaces were done on the same respective samplings. For the event *n*-gram sizes of the Γ_*S*_, Γ_*S*+*T*_ and Γ_*ST*_ feature spaces we used a fixed *n*-gram size of *n* = 2, which yielded the overall best results. We also tested using multiple values of *n* simultaneously, as e.g. described in [65]. However, the performance increase was only minimal. As a convention, all results are listed as the mean and standard deviation in percent.

#### Evaluation of the individual MCP and MCD classifiers

**Fig 3:**
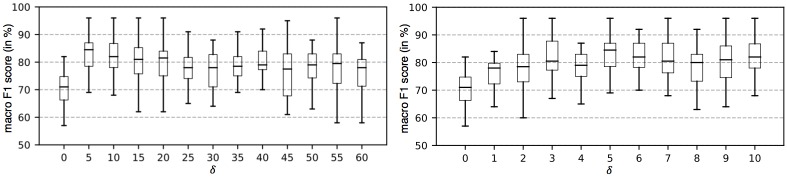
MCP evaluation on MFC data for Γ_*ST*_: F1 scores (*macro*) in% for ranges of *δ* ∈ [0, 60] (left) and *δ* ∈ [0, 10] (right).

The purpose of the first set of experiments is to find the best performing learning method for all feature spaces, s.t. we can restrict the further experiments to this learning method, allowing to focus on the feature space analyses. Since Γ_*ST*_ is further parametrized by *δ*, we start with analyzing the impact of different values of *δ* on the MCP classification performance of Γ_*ST*_ using the SVM classifier. To obtain more reliable parameter values, the calibration is conducted using solely the MCP, and not the combined MCP-MCD system. The mean sample length in the MFC data set is 44.11 events, with a standard deviation of 13.51 events. Since *δ* encodes the positional variance of the matched *n*-grams within the projected sequence, it does not make sense to increase its size beyond a value of *δ* = 60, at which the complete average sequence length is covered. Since we expect a high importance of a similar positioning of the matched *n*-grams, we expect better results for smaller values of *δ*. The left plot in Fig 3 shows the results for *δ* ∈ [0, 60]. As expected we achieve the best results with a value of *δ* ≤ 10. For that reason we focused stronger on the range of *δ* ∈ [0, 10], which is illustrated in the right plot in Fig 3, allowing to further reduce the selection of an optimal value down to *δ* = 5. As this value allows a positional variance of ±5 events on *S*^*M*^, it can additionally be explained by the circumstance that event sub-sequences, which are crucial to the protocol, like the call setup (also illustrated in Table 2) take around 10 events in the *s*_2*C*_ representation, requiring any sequence to match the contained events.

Now that we know a proper setting of *δ* we can conduct a comparative evaluation of all MCP classifiers on the MFC data set, using all feature spaces. The results are shown in Table 5. Of those learning methods which are widely used in the field of process mining, and which have been applied on Γ_*qT*_ and Γ_qT*_, the decision tree showed the overall best performance, specifically on Γ_qT*_, i.e. the original sequence representation. However, both the Markov and the LSTM classifier achieve an improved performance on the temporally enriched Γ_*qT*_ feature space. When compared to the performance on the other feature spaces though, all of those approaches commonly used in the field of process mining are clearly outperformed by the other feature spaces and learning methods. While this was to be expected, given that the process mining approach, and specifically deep learning approaches like LSTM usually require much larger training data sets, also the lack of the additionally included concrete temporal information is highly relevant, since they are even outperformed by Γ_*T*_, which only contains strongly reduced structural information about the data. Hence the SVM classifier performs best on all feature spaces, outperforming the otherwise widely used MLP, as well as the KNN approach. For those reasons we are using it for the remaining experiments. The results of the SVM classifier also show, that in the optimal scenario in which all *MC* are known, good results can already be achieved without using the proposed *θ*_*db*_ system calibration.

**Table 5:** Individual evaluation results of MCP and MCD on MFC data (%).

MCP		F1 Score (*macro*)	Precision (*macro*)
Γ_qT*_	Markov	30.90±8.06	34.62±10.97
	DCT	**52.58**±10.12	**56.03**±11.26
	LSTM	45.36±11.78	49.25±13.42
Γ_*qT*_	Markov	33.57±6.91	34.22±9.18
	DCT	48.89±9.74	52.45±11.44
	LSTM	47.38±7.85	52.55±8.14
Γ_*T*_	KNN	77.57 ±9.05	80.27 ±9.13
	MLP	80.94 ±6.83	83.55 ±6.98
	**SVM**	**84.72** ±7.34	**87.33** ±6.65
Γ_*S*_	KNN	77.51 ±8.89	81.79 ±7.96
	MLP	83.07 ±7.00	85.58 ±6.41
	**SVM**	**83.60** ±9.01	**86.17** ±8.51
Γ_*S*+*T*_	KNN	79.10 ±8.14	82.44 ±7.89
	MLP	84.27 ±7.81	87.08 ±7.24
	**SVM**	**85.25** ±7.17	**87.67** ±6.83
Γ_*ST*_	KNN	77.67 ±8.40	79.93 ±8.31
	MLP	78.53 ±8.31	82.03 ±7.55
	**SVM**	**83.67** ±7.13	**85.70** ±6.77
MCD		F1 Score (*macro*)	Precision (*macro*)
Γ_*T*_	SVM	62.91 ±19.43	64.54 ±20.33
Γ_*S*_		63.33 ±21.72	70.68 ±23.85
Γ_*S*+*T*_		65.53 ±20.64	72.57 ±22.21
Γ_*ST*_		58.63 ±25.17	76.72 ±28.09

For the MCD evaluation only the SVM classifier is evaluated. This decision was taken as it shows the most robust behavior in the MCP evaluation. The results are shown at the bottom of Table 5. Obviously discriminating the *MC* and ¬*MC* is harder than separating the *MC* in the MCP setting, which is to be expected, as the ¬*MC* samples are very heterogenous. However, using semi-supervised learning via a One-Class SVM to model each *MC*_*i*_ against the ¬*MC* performed even worse. Since all other learning methods also performed much worse, their results have been omitted to maintain a proper readability of this section. Of all feature spaces Γ_*S*+*T*_ performs best, while the specifically crafted Γ_*ST*_ feature space is slightly outperformed by all other feature spaces. While one might think that Γ_*ST*_ does not look that promising yet, the combined classifier evaluation will show, that it performs better than its competitors in the final system layout, when the effective recall becomes relevant.

#### Evaluation of the combined classifiers

**Fig 4:**
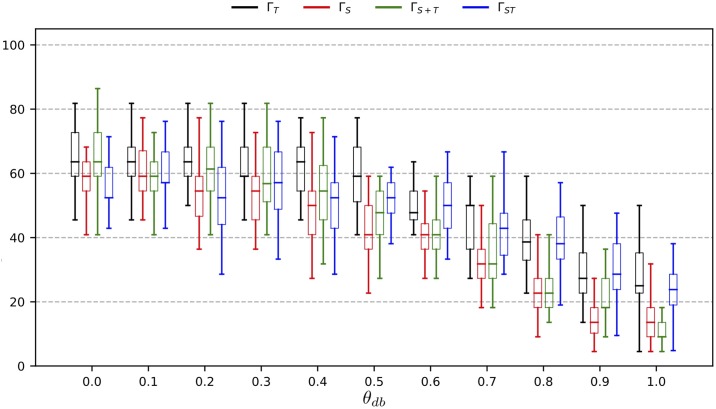
Percentage of *MC* samples in the *High*-confidence set, for *θ*_*db*_ ∈ [0, 1.0], for Γ_*T*_, Γ_*S*_, Γ_*S*+*T*_ and Γ_*ST*_.

Now that we established some understanding of the performance of the individual MCD and MCP classifiers, we will now evaluate for each qualified feature space its combined classification performance, also integrating the previously described confidence ratings. We do this to find the feature space which has the highest precision, at the highest possible effective recall, which is highly relevant for an effective system in the practical application. Achieving high precision predictions means that we can trust the results to be correctly classified and to not contain any samples of ¬*MC* falsely being classified as an *MC* sample. And getting the high effective recall means, we get this predictive behavior for a larger portion of the *MC* samples that are actually contained in the test set. This aspect is illustrated in Fig 4, which shows the percentage of *High*-confidence samples of *MC* to all samples of *MC* in the test set, under a shifting parameter *θ*_*db*_ ∈ [0, 1.0]. The more *θ*_*db*_ is increased, the less samples of *MC* are actually contained in the *High*-confidence subset, reducing the potential effective recall.

**Fig 5:**
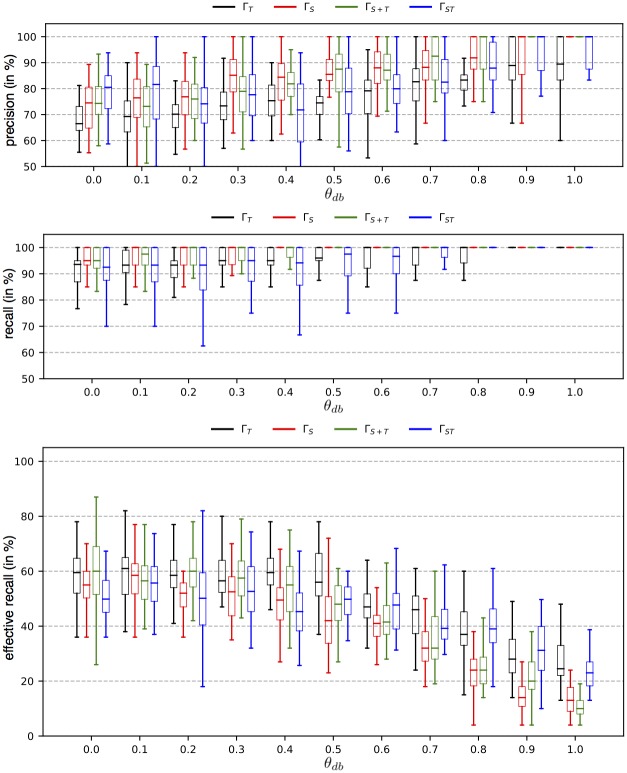
Combined classification results on MFC data: Precision, recall, effective recall (from top to bottom) in % for *θ*_*db*_ ∈ [0, 1.0], for Γ_*T*_, Γ_*S*_, Γ_*S*+*T*_ and Γ_*ST*_.

For comparing the combined classification performance of the different feature spaces with the SVM classifier, we need to select values of *θ*_*db*_ representing practically relevant values of precision and recall, which are similar for the respective feature spaces. Fig 5 contains the precision, recall and the effective recall of the classification results for *θ*_*db*_ ∈ [0, 1.0]. The precision starts to reach 100% for most classifiers at *θ*_*db*_ = 0.8. At this value also the recall reaches the maximum of 100%. When looking at the concrete values of mean and standard deviation, shown in Table 6, we see that the recall can not be further increased, and that the corresponding precision can be selected s.t. it is around 93% for Γ_*ST*_, Γ_*S*+*T*_ and Γ_*S*_. Since further increasing the precision would not further increase the recall, and 93% is already a reasonable system precision, we will use this value and the respective settings of *θ*_*db*_ for the further analyses. Thus we are using *θ*_*db*_ = 0.8 for Γ_*S*_ and Γ_*S*+*T*_, and *θ*_*db*_ = 0.9 for Γ_*ST*_. In this respect, the performance of Γ_*T*_ was not sufficiently high to achieve similar values of precision and recall, which is why we used *θ*_*db*_ = 1.1 there, achieving relatively close values for further analyses.

**Table 6:** Combined evaluation results on MFC data (in %).

	*θ*_*db*_	F1 Score	Precision	Recall	Eff. Recall
Γ_*T*_	0.0	76.37 ±7.90	69.09 ±8.22	91.12 ±7.73	61.57 ±9.39
	1.1	90.71 ±13.28	87.40 ±16.75	98.44 ±7.67	17.25 ±7.49
Γ_*S*_	0.0	80.82 ±7.18	73.74 ±8.42	95.32 ±5.55	57.94 ±10.17
	0.8	95.01 ±6.26	92.65 ±8.92	99.90 ±1.00	25.05 ±7.98
Γ_*S*+*T*_	0.0	81.23 ±7.65	75.28 ±8.09	95.02 ±5.54	60.50 ±12.77
	0.8	95.64 ±5.54	**93.51** ±8.13	**100.0** ±0.0	**26.27** ±8.64
	0.9	97.36 ±4.86	95.97 ±7.39	100.0 ±0.0	20.38 ±8.09
Γ_*ST*_	0.0	81.52 ±8.83	77.74 ±10.97	91.73 ±7.35	50.84 ±8.28
	0.8	91.72 ±8.29	88.29 ±10.34	99.17 ±4.49	39.88 ±8.81
	0.9	95.33 ±6.32	**93.11** ±9.16	**100.0** ±0.0	**31.79** ±10.33
E	0.0	81.98 ±8.33	78.53 ±9.07	90.10 ±9.40	55.78 ±10.73
	0.8	88.74 ±13.49	89.76 ±13.65	90.72 ±13.58	23.59 ±7.76
	0.9				

Now we need to evaluate which feature space offers the best effective recall, i.e. the fraction of *MC* samples that can be recovered from the set of test samples, with those high values of precision and recall previously described. At their respective values of *θ*_*db*_, Γ_*S*_ has the lowest effective recall of 25.05%, followed by Γ_*S*+*T*_ with 26.27%, and Γ_*ST*_ with an effective recall of 31.79%. Thus Γ_*ST*_ produces a precision performance similar to both Γ_*S*+*T*_ and Γ_*S*_, while achieving a 5.5% higher effective recall than Γ_*S*+*T*_, and a 6.7% higher effective recall than Γ_*S*_. This means, we can get for 31.79% of the *MC* samples in the test set a correct prediction with 93.11% precision and 100% recall when using Γ_*ST*_, compared to 26.27% effective recall, 93.51% precision and 100% recall when using Γ_*S*+*T*_, and even worse when using Γ_*S*_. These results are highly relevant in practice, as they effectively allow filtering near-certain from uncertain predictions with a very high precision. These results are also highlighting the effectiveness of both combined feature spaces, with a significant advantage for the Γ_*ST*_ feature space. In that regard both structural-temporal feature spaces Γ_*S*+*T*_ and Γ_*ST*_ outperform Γ_*S*_ and Γ_*T*_: Whereas Γ_*T*_ has a relatively good effective recall, but a relatively low precision, Γ_*S*_ has an acceptable precision, but a low effective recall. This renders both feature spaces less practically relevant than their combined counter parts, highlighting the relevance of combined structural-temporal feature spaces.

The dimensions most relevant for the respective classification results in this use case were security handshake events, followed by the existence of events representing a successful response to the most relevant key protocol states, like a successful radio bearer setup. As we saw in the MCP evaluation, the temporal features are also relevant and utilized in both combined feature spaces. The Γ_*ST*_ feature space also has an advantage here, as its *S*^*M*^-based feature space allows locating the concrete positions and structural-temporal properties of the relevant events within the sequence, which are in the evaluation often identified as responses occurring too late in the sequence, or security mode negotiations at anomalous sequence positions. All of this can then be used to obtain deeper insights into the data, which can help manual analysts to limit the number of causes for this specific failure class.

Due to their potential in combining classifiers of different features spaces, we also evaluate the classification performance of an ensemble method [66], namely the ensemble classifier E, which could potentially further optimize precision and effective recall. It predicts the combined classification results by using a majority voting over the predicted labels of Γ_*S*_, Γ_*S*+*T*_ and Γ_*ST*_. Table 6 shows its results when using their default trained models of *θ*_*db*_ = 0.0, and for their optimized decision boundary models, using *θ*_*db*_ = 0.8 for Γ_*S*_ and Γ_*S*+*T*_, and *θ*_*db*_ = 0.9 for Γ_*ST*_ respectively. Γ_*T*_ has not been used due to its lower performance. When using the default models at *θ*_*db*_ = 0.0, the ensemble classifier in fact achieves the best precision at the cost of the effective recall, an effect similar to the trade-off of *θ*_*db*_. For the optimized models of *θ*_*db*_ ∈ {0.8, 0.9} the ensemble classifier achieved worse results though.

#### Significance analysis

To further substantiate the results of the previous section, we conduct significance tests on both of our theoretical hypotheses, namely that the combined feature spaces Γ_*S*+*T*_ and Γ_*ST*_ outperform the base feature spaces Γ_*T*_ and Γ_*S*_ in terms of effective recall at similar precision (Hypothesis *H*^*A*^), and that the more complex combined feature space Γ_*ST*_ outperforms the simpler combined feature space Γ_*S*+*T*_ under the same premises (Hypothesis *H*^*B*^). For the formulation of the hypotheses we denote *θ*_*er*_ as the minimal effective recall.

For hypothesis *H*^*A*^ the null hypothesis $H_{0}^{A}$ is defined as follows: When using Γ_*S*_ or Γ_*T*_ for achieving a test set precision mean of 93%, a fraction of *p*_0_ samplings have an effective recall ≥ *θ*_*er*_. The alternative hypothesis $H_{1}^{A}$ is then defined as follows: When using Γ_*ST*_ or Γ_*S*+*T*_ for achieving a test set precision mean of 93%, a fraction of $\hat{p}$ samplings have an effective recall ≥*θ*_*er*_. Now we can formulate the question for hypothesis *H*^*A*^: Is there sufficient evidence at the *α* = 0.05 level to conclude that the effective recall for the high precision classification performance is increased, when using one of the combined feature spaces Γ_*ST*_ or Γ_*S*+*T*_ instead of one of the individual feature spaces Γ_*T*_ or Γ_*S*_? And at which minimal effective recall *θ*_*er*_ does this hold? The results for the minimal *θ*_*er*_, at which we can reject $H_{0}^{A}$ in favor of $H_{1}^{A}$ (i.e. above which *p* ≤ *α* always holds for the resulting *p*-values) are shown in Table 7, for each pair of base and combined feature space, as calculated on the same sampling that have also been used for the previous combined MFC evaluation. We can see that $H_{0}^{A}$ can be rejected for Γ_*T*_ for values of *θ*_*er*_ ≥ 5%, i.e. for nearly all values of *θ*_*er*_, excluding those which do not occur in the combined feature spaces due to their generally higher effective recall. For Γ_*S*_, $H_{0}^{A}$ can be rejected for *θ*_*er*_ ≥ 9% for Γ_*ST*_, and for *θ*_*er*_ ≥ 14% for Γ_*S*+*T*_. This means Γ_*ST*_ is better for a larger number of samplings, while Γ_*S*+*T*_ starts outperforming Γ_*S*_ later—both of which is also relevant for hypothesis *H*^*B*^.

**Table 7:** Values of minimal *θ*_*er*_ to reject hypotheses $H_{0}^{A}$ and $H_{0}^{B}$ at *α* = 0.05.

		Γ_*S*+*T*_	Γ_*ST*_
*H*^*A*^	Γ_*T*_	5%	5%
	Γ_*S*_	14%	9%
*H*^*B*^	Γ_*S*+*T*_	-	30%

For hypothesis *H*^*B*^ the null hypothesis $H_{0}^{B}$ is defined as follows: When using Γ_*S*+*T*_ for achieving a test set precision mean of Â 93%, a fraction of *p*_0_ samplings have an effective recall ≥*θ*_*er*_. The alternative hypothesis $H_{1}^{B}$ is then defined analogous: When using Γ_*ST*_ for achieving a test set precision mean of Â 93%, a fraction of $\hat{p}$ samplings have an effective recall ≥*θ*_*er*_. The question for hypothesis *H*^*B*^ is then: Is there sufficient evidence at the *α* = 0.05 level to conclude that the effective recall for the high precision classification performance is increased, when using the complex combined feature space Γ_*ST*_ instead of the simpler combined feature space Γ_*S*+*T*_? And at which minimal effective recall *θ*_*er*_ does this hold? As shown in Table 7, $H_{0}^{B}$ can be rejected for all values of *θ*_*er*_ ≥ 30%, showing that Γ_*ST*_ indeed outperforms Γ_*S*+*T*_, a fact that is further strengthened by the performance advantage of Γ_*ST*_ over Γ_*S*+*T*_, as shown for hypothesis *H*^*A*^.

**Fig 6:**
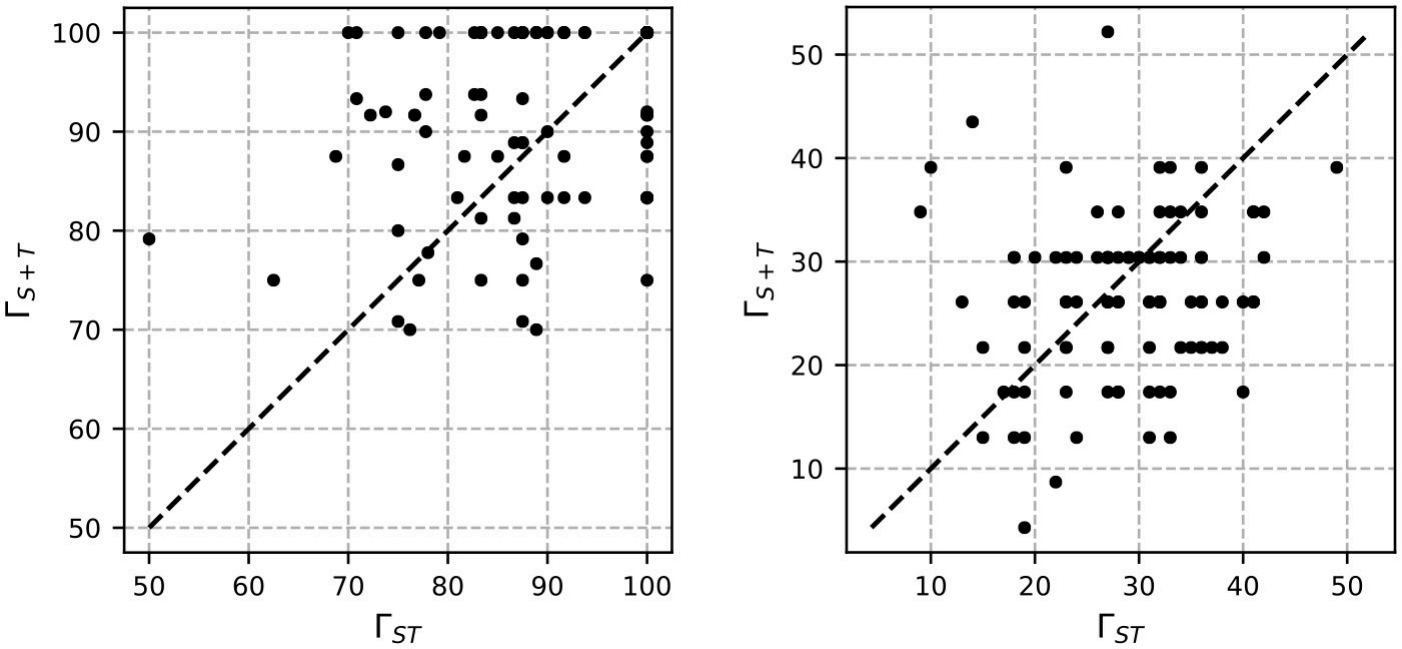
Precision (left) and effective recall (right) of Γ_*ST*_ against Γ_*S*+*T*_(in %).

We will now further elaborate hypothesis *H*^*B*^ by analyzing the distribution of precision and effective recall, when using either feature space on the same test sets. The results of this performance variance analysis are shown in Fig 6. As previously stated, and shown in Table 6, we conducted the significance tests on SVM classification models calibrated for an average precision ≥ 93%. As shown in the left plot of Fig 6, the models of both Γ_*S*+*T*_ and Γ_*ST*_ show similar performance distributions, with a slight advantage for Γ_*S*+*T*_, due to its slightly higher average precision of 93.51%, compared to the 93.11% of Γ_*ST*_. However, as the right plot shows, the effective recall on the same test sets is much balanced towards Γ_*ST*_, clearly supporting hypothesis *H*^*B*^. As a result these analyses support our conclusion that Γ_*ST*_ is the most advantageous feature space in the discussed use case.

### Experiments on AFC data

Due to the already discussed shortcomings of the AFC data set properties, we are only interested to see, whether the MCP are capable of discriminating the model classes of the AFC data set at all, and how much of a performance improvement we can expect with a larger training data set, which is possible only on the AFC data set. Similar to the class size restrictions described for the MFC evaluation, we have to ensure sufficiently large as well as similarly sized failure classes. To reflect smaller and larger training data sets, we evaluate two different setups. The first setup is defined with comparability to the MFC evaluations in mind. Hence we use *s*_#*MC*_ = 25, resulting in the 13 sufficiently sized failure classes listed in Table 1. In the second setup, selecting *s*_#*MC*_ = 100 allows for a larger training data set, resulting in 4 sufficiently sized failure classes. For defining the Γ_*ST*_ features we used all 3,264 sequences as model sequences *S*^*M*^, resulting in a feature space of 144,938 dimensions after redundancy-based dimensionality reduction.

Table 8 shows the results of the MCP evaluations on the AFC data set. Due to the differences in the MFC and the AFC data, we expected a worse classification performance than on the MFC data, which indeed occurs. However, for the larger sets of training data with *s*_#*MC*_ = 100 the results are largely improved, which shows, that the AFC data set still contains a sufficient number of discriminative features to enable classification. This also documents the potential for an equally increased classification performance on the MFC data, once more class-wise training data is there available as well—which also applied to the general use case of similar classification problems.

**Table 8:** Individual evaluation results of MCP (SVM) on AFC data (in %).

MCP	*c*_#*MC*_	F1 Score (*macro*)	Precision (*macro*)
Γ_*S*+*T*_	25	46.84 ±4.71	49.17 ±6.17
Γ_*ST*_		42.00 ±5.63	43.97 ±6.12
Γ_*S*+*T*_	100	71.15 ±4.09	72.08 ±4.17
Γ_*ST*_		62.90 ±6.05	63.67 ±6.08

## Conclusion

This paper addresses theoretical and practical issues, relevant when analyzing real-time log data of structural, temporal processes using specific structural-temporal feature spaces for solving classification problems on mobile communication failure data. On the theoretical side we present an analysis of structural and temporal data properties, specifically on the discussed format of mobile communication data. We introduce and discuss novel individual and combined feature spaces utilizing those properties to obtain a good data representation. We conduct a comparative performance evaluation of these feature spaces against feature spaces and on a range of classification methods, all of which are commonly used in related work. We also show in various evaluations and via hypothesis testing that both of our combined temporal structural feature spaces Γ_*S*+*T*_ and Γ_*ST*_ outperform their competition counterparts from the research fields of sequence learning and process mining, and that the novel Γ_*ST*_ feature space excels in classification performance when compared to all other approaches, including an ensemble method. On the practical side we propose a system for the detection and prediction of classes of pre-defined sequence behavior, applied on the use case of the automatic classification of mobile communication failures using the proposed feature spaces and supervised learning, for which we also show how to maximize its classification precision and effective recall via a calibration procedure. We highlight the importance of properly labeled training data, for which we show that our proposed Γ_*ST*_ feature space is able achieve a highly trustworthy precision of more than 93% while having the advantage of an up to 6.7% higher effective recall than the other feature spaces. These results are highly relevant in practice, as they effectively allow separating reliable from unreliable predictions. With the higher effective recall more reliable predictions can be obtained, further reducing the costs of otherwise unfeasible manual analysis processes. We also discussed approaches to further improve those predictions.

As an outlook it could be interesting to evaluate the potential of word vector representations like those of [22] for corpora of structural-temporal data. This would not necessarily reflect the temporal data aspects, and would also require data sets much larger than currently available. However, the sequential and contextual aspects of the event relations could potentially be covered, which could help improving the interpretability of the internal process relations, as well as the overall classification performances. Additionally—and despite the differences in the described data properties and the problems to be solved—a prospective comparison of the proposed features with those of the field of process mining and business intelligence analysis will be highly relevant for future research, e.g. by reformulating our failure sequence classification problem as one of predicting the next events and remaining time. To achieve a more focused scope, this manuscript deliberately limits the comparative evaluations to specific feature representations and learning methods. While those have been chosen based on their use in related work, the primary selection criterion was to allow comparing and incorporating the proposed structural-temporal features. Hence the evaluation of otherwise closely related feature representations (e.g. the complex sequence encoding of [35]) on network communication data sets need to be part of our future work as well, potentially enabling an extension of current process mining approaches to also cover complex temporally aware multi-class problems on event-based communication data like the one discussed here.

## Nomenclature

**Table:** 

***MC***_***i***_	**A model class, i.e. a sequence class sufficiently sized to model a classifier**
*s*_#*MC*_	Configuration parameter for the number of samples in this model class
|*s*|	Number of events in a sequence s
¬*MC*	non Model Class, containing samples of insufficiently sized classes
MCP	Model Class Predictor
MCD	Model Class Detector
Γ_*qT*_	Quantized temporal feature space
Γ_*T*_	Temporal feature space
Γ_*S*_	Structural feature space
Γ_*S*+*T*_	Concatenated structural temporal feature space
Γ_*ST*_	Structural temporal feature space
*S*	Set of sequences *s*
*S*^*M*^	Set of model sequences *s*^*M*^, defining the Γ_*ST*_ feature space model
Θ_*MCD*_	Confidence rating based on the MCD
Θ_*db*_	Confidence rating based on the decision boundaries of the MCP
*θ*_*db*_	Calibration parameter for the MCP decision boundaries

## Appendix

### Relevant dimension estimation

**Fig 7:**
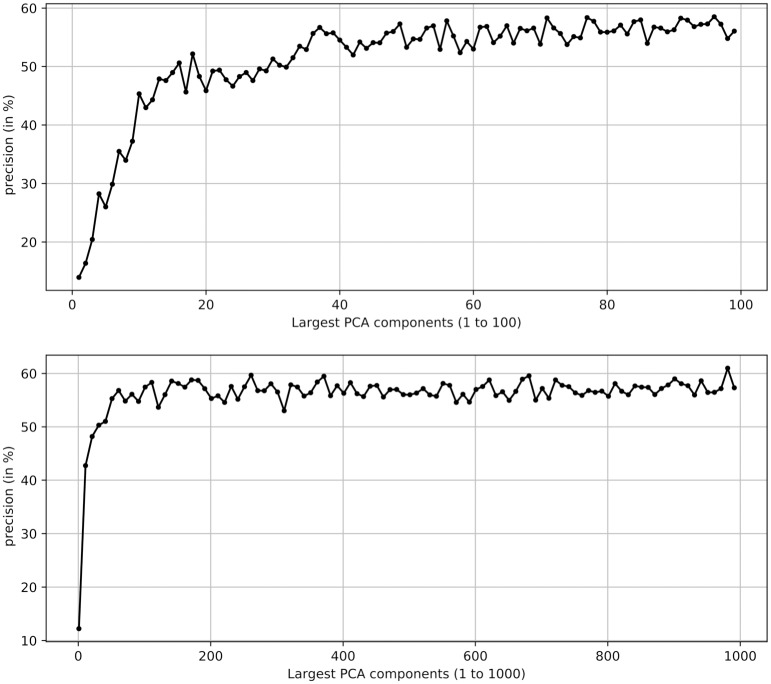
Results over the first 100 (top) and 1.000 (bottom) dimensions of the largest PCA components using a condensed set of Γ_*ST*_ features.

Due to the projection on the set of model sequences *S*^*M*^, the resulting Γ_*ST*_ feature space can become very large. To estimate the potential for the application of a dimensionality reduction approach, we conducted additional experiments using Γ_*ST*_ and the model class predictors (MCP) on the MFC data, to achieve an empirical estimation of the number of relevant dimensions, similar to the analyses conducted in [62]. However, the sparsity of the Γ_*ST*_ feature space, together with the limited size of the analyzed data sets makes it hard to analyze the full set of dimensions with a sufficient statistical robustness. To achieve statistically more robust results, the number of base dimensions of Γ_*ST*_ was reduced to those 5.000 dimensions with the largest variances, selected from a filtered set of those dimensions that had a density > 70%. For those dimensions a PCA analysis was conducted repetitively, to extract the *k* largest PCA components in a range of 1 to 1.000. We then projected the train and test samples onto the dimensions defined by those *k* largest PCA components to achieve a feature representation with a reduced dimensionality. This allowed us to train and test a linear classifier on each of those projected sets, obtaining the results for the largest 100 and 1.000 PCA dimensions respectively, as shown in Fig 7.

As can be seen, the performance slowly increases, as long as additional dimensions are added to the utilized feature space. Although the variance is still relatively high, we already achieve a convergence at about 100 dimension. The variance starts to decrease from 400 dimensions on. This means, that for this set of filtered dimensions, a reduction to the 400 largest PCA dimensions can be achieved without loosing much of the precision of the base feature set. Due to the restrictions mentioned above, the results achieved on this limited set of dimensions do not represent the full set of features. This also explains, why the results are lower than those presented in Table 5. Despite these restrictions, the results allow establishing the hypothesis that a much lower number of dimensions may be sufficient when using the Γ_*ST*_ feature space. This hypothesis needs to be tested on larger data sets in the future.

## Supporting information

S1 Data The MFC and AFC data sets. Each communication sequence is provided in an individual file, containing all relevant data and using anonymized failure classes, protocol identifiers and event identifiers.
(ZIP)
